# Homocysteine Homeostasis and Betaine-Homocysteine S-Methyltransferase Expression in the Brain of Hibernating Bats

**DOI:** 10.1371/journal.pone.0085632

**Published:** 2013-12-23

**Authors:** Yijian Zhang, Tengteng Zhu, Lina Wang, Yi-Hsuan Pan, Shuyi Zhang

**Affiliations:** Laboratory of Molecular Ecology and Evolution, Institute for Advanced Studies in Multidisciplinary Science and Technology, East China Normal University, Shanghai, China; CSIRO, Australia

## Abstract

Elevated homocysteine is an important risk factor that increases cerebrovascular and neurodegenerative disease morbidity. In mammals, B vitamin supplementation can reduce homocysteine levels. Whether, and how, hibernating mammals, that essentially stop ingesting B vitamins, maintain homocysteine metabolism and avoid cerebrovascular impacts and neurodegeneration remain unclear. Here, we compare homocysteine levels in the brains of torpid bats, active bats and rats to identify the molecules involved in homocysteine homeostasis. We found that homocysteine does not elevate in torpid brains, despite declining vitamin B levels. At low levels of vitamin B6 and B12, we found no change in total expression level of the two main enzymes involved in homocysteine metabolism (methionine synthase and cystathionine β-synthase), but a 1.85-fold increase in the expression of the coenzyme-independent betaine-homocysteine S-methyltransferase (BHMT). BHMT expression was observed in the amygdala of basal ganglia and the cerebral cortex where BHMT levels were clearly elevated during torpor. This is the first report of BHMT protein expression in the brain and suggests that BHMT modulates homocysteine in the brains of hibernating bats. BHMT may have a neuroprotective role in the brains of hibernating mammals and further research on this system could expand our biomedical understanding of certain cerebrovascular and neurodegenerative disease processes.

## Introduction

Homocysteine is a sulfur-containing amino acid and a risk factor involved in cerebrovascular and neurodegenerative diseases [1–5]. The elevation of homocysteine may result in the dysfunction of endothelial and smooth muscle cells in the vascular wall. Endothelial injuries such as the inhibition of cellular binding sites for tissue plasminogen activator [6], decreasing expression of thrombomodulin [1,7], and production of endoplasmic reticulum stress and growth arrest [8] are observed in hyperhomocysteinemic animals. The effect of hemostasis, induced by homocysteine, promotes blood clotting and reduces fibrinolysis [9]. Elevated homocysteine impairs smooth muscle cells by inducing a proliferative state, and migration from the media to the intima of the vessel [10]. These homocysteine-induced changes may initiate the pathogenesis of atherosclerosis [10] which can lead to vascular diseases in the brain. Elevated homocysteine expression is also known to play an important role in neurodegenerative diseases including brain atrophy, dementia and cognitive impairment [4,11,12]. For example, homocysteine is elevated in patients with confirmed Alzheimer’s disease [11,13,14]. Homocysteine causes excitotoxic and oxidative injury to hippocampal neurons in cell cultures and *in vivo* [15], and hyperhomocysteinemia due to dietary folate deficiency endangers dopaminergic neurons in models of Parkinson’s disease [16]. 

**Figure 1:**
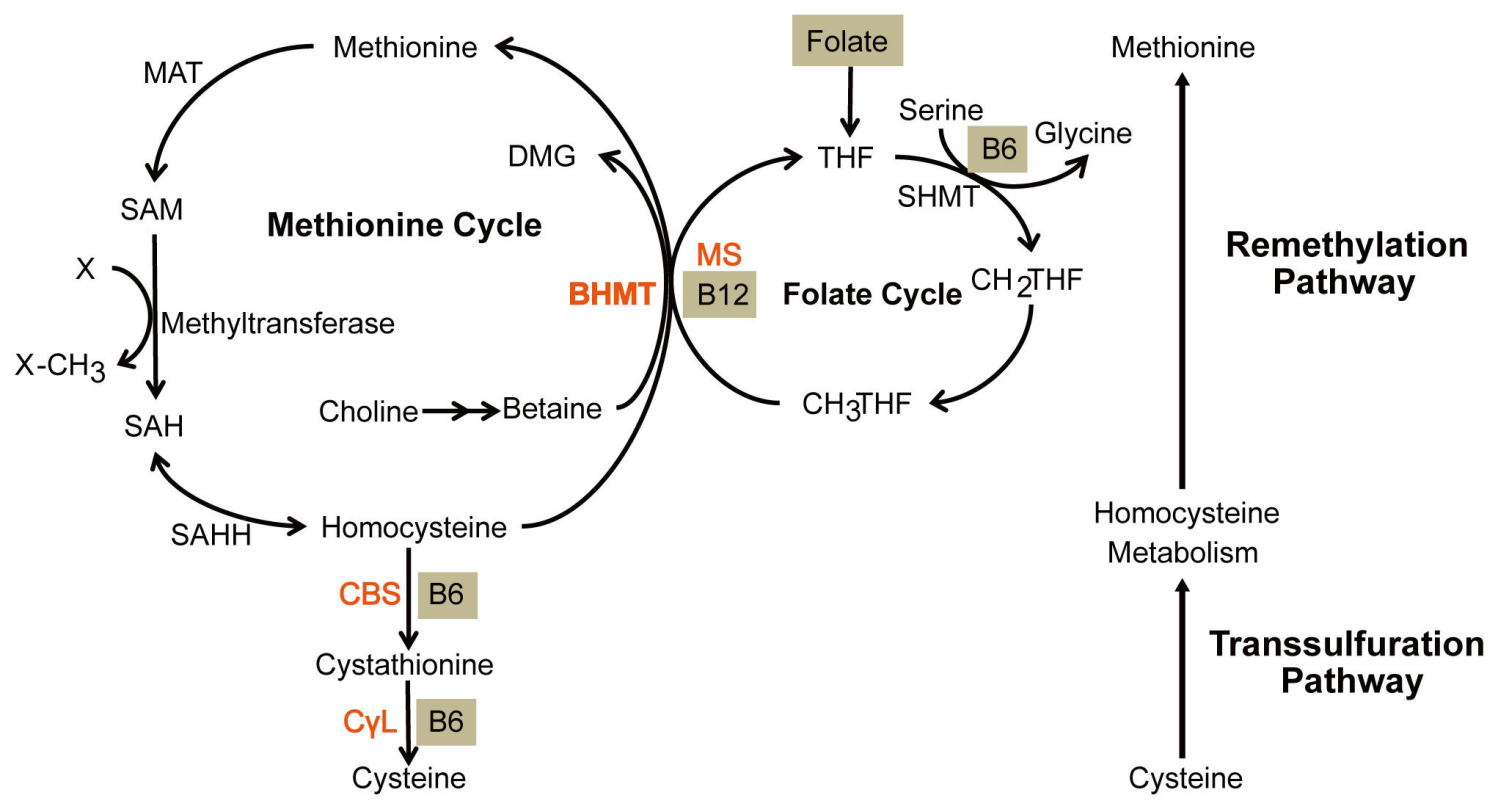
Homocysteine metabolism. Homocysteine is metabolized to methionine by remethylation and cystathionine by transsulfuration. Coenzymes are shown in gray. BHMT, betaine-homocysteine S-methyltransferase; DMG, dimethylglycine; MAT, methionine adenosyltransferase; SAM, S-adenosylmethionine; SAH, S-adenosylhomocysteine; SAHH, S-adenosylhomocysteine hydrolase; MS, methionine synthase; THF, tetrahydrofolate; SHMT, serine hydroxymethyltransferase; CH2THF, methylene tetrahydrofolate; CH3THF, methyl tetrahydrofolate; CBS, cystathionine β-synthase; CγL, cystathionine γ-lyase.

Homocysteine metabolism involves remethylation or transsulfuration (Figure 1) [17,18]. During remethylation, homocysteine is methylated to methionine by methionine synthase (MS, EC 2.1.1.13), which is ubiquitous, or by betaine-homocysteine S-methyltransferase (BHMT, EC 2.1.1.5), whose expression is mainly restricted to the liver and kidney [19]. During transsulfuration, homocysteine is irreversibly converted into cysteine by cystathionine β-synthase (CBS, EC 4.2.1.22) and cystathionine γ-lyase (CγL, EC 4.4.1.1) in the liver, kidney, pancreas and small intestine [17]. Both the remethylation and transsulfuration of homocysteine involve B vitamins: methionine synthase requires folic acid and vitamin B12 as substrates or cofactors, and cystathionine β-synthase is a vitamin B6-dependent heme protein. Inadequate levels of one or more B vitamins contribute to elevated homocysteine levels and neurological damage [20,21]

Some mammals reduce body temperature, metabolic rate and other physiological processes during hibernation, which is an adaptive strategy in response to winter [22–24]. Hibernation involves fasting [22] and may therefore be a critical time during which the metabolism of homocysteine is inhibited because of B vitamin deficiency. How fasting mammals avoid elevated homocysteine and its negative neurological impacts remains unknown.

Bats are the second most abundant mammal species on earth and the majority of microbats hibernate [25]. Recent research has revealed protective mechanisms in the brain tissue of hibernators [26–28], and hibernating species are emerging as ideal research models for neurological disease [29–32]. In this study, we investigate brain tissue in torpid and active Rickett’s big-footed bats (*Myotis ricketti*) to determine patterns of homocysteine homeostasis and metabolism during fasting. We aimed to (1) identify whether BHMT, MS and CBS are involved in homocysteine metabolism in the brain and describe expression patterns in different brain regions, and (2) give the likely role and importance of these enzymes in homocysteine regulation, describe the nature of selection operating on underlying genes. While expanding our understanding of adaptations to hibernation and fasting, this research will also contribute to the broader biomedical prevention and treatment of human cerebrovascular and neurodegenerative diseases.

## Materials and Methods

### Ethics statement

All procedures involving the capture of bats and collection of samples were carried out in strict accordance with the Guidelines and Regulations for the Administration of Laboratory Animals (Decree No. 2, the State Science and Technology Commission of the People’s Republic of China on November 14, 1988) and were approved by the Animal Ethics Committee at East China Normal University (ID no: AR2012/03001).

### Collection of animals and tissues

Hibernating male *M. ricketti* (n = 14) were captured from Fangshan Caves (39°48′ N, 115°42′ E), Beijing, China. Seven animals were immediately euthanized and their core body (T_b_) and surface (T_a_) temperatures were 13 ± 2°C and 9 ± 1°C, respectively. Seven *M. ricketti* were euthanized 48 h after arousal and their core body temperature (T_b_) was 35 ± 2 °C. Non-hibernating *Rousettus leschenaultii* (n = 4) were captured in Mashan county (23°55′ N, 108°26′ E), Guangxi, China. These animals (T_b_ = 35 ± 2 °C) were sacrificed 48 h after capture by cervical dislocation. Male rats (n = 4) were obtained from Sino-British Sippr/BK Lab Animal Ltd (Shanghai, China). Brain tissue was rapidly removed and collected in 2 ml cryotubes and stored at -80°C.

### B vitamins, homocysteine and betaine assays

Vitamin B6, vitamin B12, folate, homocysteine and betaine levels in brain tissue and blood were determined using vitamin B6 (TSZ-E80574), vitamin B12 (TSZ-E80166), folate (TSZ-E80542), homocysteine (TSZ-E30175) and betaine (TSZ- S25623) assay kits (Yanyu Chemical Reagent Inc., Shanghai) according to the manufacturer’s instructions. Brain tissue (20 mg) was homogenized in 200 μl PBS (137 mM NaCl, 2.7 mM KCl, and 10 mM Na_2_HPO_4_). The homogenate was then centrifuged at 14,000 xg, 4°C for 15 min, and the supernatant was assayed for B vitamins, homocysteine and betaine levels [33]. Blood was obtained by cardiac puncture and centrifuged for 15 min, and plasma was frozen at -80°C for use. The 96-well microplate was incubated at 37°C for 30 min each with sample and HRP-reagent. After adding A and B developer buffers, the reaction last 10 min, and was stopped using stop solution. OD_450_ was measured within 15 min. B vitamins, homocysteine and betaine concentrations in samples were quantified by comparing OD_450_ values against those of known concentration. Results (mean ± SD) are from three separate experiments (n = 3 per group) and analyzed using Student’s t-tests (two-tailed). A P value < 0.05 was considered significant.

### Western blots

Brain proteins were extracted from bats and rats. The tissue was homogenized by lysis buffer (0.22 M Tris-HCl (pH 6.8), 8.8% SDS, 44.4% glycerol) and centrifuged at 12,000 xg, 4°C for 10 min. The supernatant was heated at 100°C for 10 min and then used for western blotting. Brain proteins of the rat were used as a positive loading control. Proteins in each sample (10 μg/lane) were separated by 10% SDS-PAGE and then transferred onto 0.2 μm PVDF membranes (Millipore, USA) with an electro-blotting apparatus (GenePure, Taiwan). The PVDF membranes were blocked in blocking solution containing 5% skim milk and 1% BSA at 4°C for 12 h, and then reacted with a series of primary antibodies including anti-BHMT (1:2500), anti-MS (1:250), and anti-CBS (1:1000). The anti-BHMT (ab96415) and anti-MS (ab9209) were acquired from Abcam Corporation, and anti-CBS (sc-67154) was purchased from Santa Cruz Biotechnology, Inc. Antibodies were selected based on the ability to combine with conserved epitopes of target proteins of many mammalian species. After washing, blots were reacted with appropriate secondary antibodies and visualized according to the instructions of the Immobilon^TM^ Western Chemiluminescence HRP substrate kit (Millipore, USA). Images were captured using ImageQuant^TM^ LAS-4000 (Amersham Biosciences, USA), and detected bands were quantified with ImageQuant^TM^ TL (v 7.0, Amersham Biosciences, USA). The intensity of each band was normalized to β-actin (sc-47778, 1:5000, Santa Cruz Biotechnology Corporation). Results (mean ± SD) were calculated from four repeats and then analyzed using Student’s t-tests (two-tailed). A P value < 0.05 was considered statistically significant.

### BHMT assay

Sample preparation and BHMT activity measurement were carried out following standard protocols [34–36] with several modifications. Brain tissue (0.1 g) was homogenized in 500 μl potassium phosphate buffer (40 mM, pH 7.5) containing 1 mM DTT. The homogenate was centrifuged at 18,000 xg, 4°C for 15 min, and the supernatant fraction was used for BHMT assay. The protein concentration of each sample was assayed with the Quick Start^TM^ Bradford protein assay kit (Bio-Rad, USA) according to the manufacturer’s instructions. 

The 280 μl standard mixture contained 10 mM DL-Hcy, 3.25 mM betaine, 50 mM Tris-HCl (pH 7.5) and preparing sample (80 μl). Assays were started by transferring the tubes to a 37°C water bath for 1–2 h. Following incubation, the mixture was chilled in ice water and centrifuged at 12,000 xg, 4°C for 15 min. Phenyl isothiocyanate as the derivatization reagent, was added to the supernatant. After standing at ambient temperature for 10 min, n-hexane was used to remove organic substances, and the water-soluble substances were filtered (0.22-μm filter) for use. 

Samples were analyzed on a HPLC system equipped with separations module (Waters e2695) and a Pntulips^TM^ BP-C18 (5 μm, 4.6 x 250 mm) with a 0.8 ml/min flow rate. Methionine was monitored by a photodiode array detector (Waters 2998) with an excitation wavelength of 254 nm. HPLC data were collected and analyzed using the Great Resource Health Standard Inc. (Shanghai) database. Methionine in a sample was identified and quantified by comparing the peak retention time and peak volume of the sample to internal standard methionine. Blanks contained all of the components except for the reactants DL-Hcy and betaine, and their values were subtracted from sample values. We compared the amount of methionine to reflect brain BHMT activity in torpid and active *M. ricketti* (n = 3 per group). All samples were assayed in three repeats, and data analyzed using two-tailed Student’s t-tests. Enzyme activity is expressed as nmol/h/mg protein. 

### Immunohistochemistry

Brain tissue was fixed in 4% paraformaldehyde buffer solution for 24 h after sampling. A series of graded alcohols and xylene were used for dehydration and clearing of tissue. Brain serial-sections (6 μm) were prepared after being paraffin-embedded. Hydrogen peroxide solution (3%) was used to stop endogenous peroxidase activity after the brain sections were rehydrated, and then blocked with bull serum albumin (0.3%). After rinsing with phosphate buffer solution (PBS, 0.1 M/L), brain sections were incubated in affinity-purified primary antibody (anti-BHMT, 1:1000, abcam, ab96415) diluent overnight at 4°C. Simultaneously, normal rabbit IgG (sc-2027, 1:2000, Santa Cruz, USA) and PBS were used to replace the primary antibody in control groups. After washing with 0.1 M/L PBS, slices were reacted with corresponding secondary antibody (about 45 min at 37°C) and then antigen-antibody complexes were detected using ChemMateTM EnVison^TM^/HRP complex (including diaminobenzidine, DAB) as a peroxidase substrate (GK500705, Gene Tech). Results were visualized under an optical microscope (Lecia DM2500, Germany). All pictures were taken under the same microscope with uniform parameters after brain sections were mounted with xylene sealant.

Because bat brain atlases are not available, the mouse brain atlas [37] was used to localize the distribution of BHMT. We evaluated immunostaining for BHMT in the brains obtained from torpid and active *M. ricketti* bats (n = 3 per group). Images of the slices at 50× magnification were captured and analyzed using Image Pro Plus (Immagine Computer, Italy). Staining intensity was computed as integrated optical density (IOD) from four sections of expressed regions. The IOD was calculated from three arbitrary fields with the same area for each section. Data were analyzed using Student’s t-tests (two-tailed).

### Molecular evolution analyses and homology modeling of bat BHMT

The coding regions of *Bhmt* were sequenced for 12 bat species from seven families (Table S1), including three non-hibernating species: *Eonycteris spelaea, Rousettus leschenaultii* and *Cynopterus sphinx* (Pteropodidae); and nine hibernating species: *Taphozous melanopogon* (Emballonuridae), *Miniopterus fuliginosus* (Miniopteridae), *Pipistrellus pipistrellus* and *Myotis ricketti* (Vespertilionidae), *Artibeus lituratus* and *Leptonycteris yerbabuenae* (Phyllostomatidae), *Hipposideros armiger* and *Hipposideros pratti* (Hipposideridae), and *Rhinolophus ferrumequinum* (Rhinolophidae). The available published sequences (in Ensembl database) of two other species, *Pteropus vampyrus* (Pteropodidae) and *Myotis lucifugus* (Vespertilionidae), were also incorporated in molecular analyses. Details (BHMT accession numbers and thermal physiology) of all species are listed in Table S1.

We selected *M. ricketti* to identify sequences of *Bhmt* from the brain and liver, and found they were consistent. Some bat species do not express BHMT in brain tissue, and so all cDNA sequences are cloned from liver tissue. Following standard protocols, total RNAs were isolated from liver tissue using Trizol reagent (Invitrogen) and were reverse-transcribed to cDNA with the RNAiso Plus kit (TaKaRa). Using primers (Table S2), all PCR products were isolated and puriﬁed using the Gel Extraction kit (Qiagen), and then ligated into pGEM-T Easy Vector (Promega). Correct recombinant clones were sequenced using the Terminator kit on an ABI 3730 DNA sequencer (Applied Biosystems). 

The BHMT nucleotide sequences of 14 species were aligned using Clustal X [38]. The pairwise dN/dS ratio (ω value, non-synonymous substitution rate/synonymous substitution rate) was calculated using Swaap v1.0.3 to determine selective pressure acting on *Bhmt* [39,40]. Data were analysed using two-tailed Student’s t-tests and a P value < 0.05 was considered significant.

To determine whether amino acid residue divergence between hibernating and non-hibernating bats is related to BHMT function we simulated BHMT structure in bats. The amino acid sequences of bat BHMT were deduced from corresponding nucleotide sequences. Structure-based amino acid sequence alignments were carried out by T-Coffee (Expresso mode) to determine the amino acid residue conserved in hibernators but diverged in non-hibernators [41]. The BHMT amino acid sequence of *Rattus norvegicus* with known structure (1UMY, O09171), was selected as the template for alignment. The corresponding amino acid sequence files of *M. ricketti* were imported into SWISS-MODEL for homology modeling of BHMT structures [42]; PyMol was used to display the 3D structures and illustrate model results. 

## Results

### Vitamin B, homocysteine and betaine levels in torpid *M. ricketti*

We measured levels of vitamin B6, vitamin B12 and folate in torpid *M. ricketti* and non-torpid/non-fasting *M. ricketti*, *R. leschenaultia* and rats. We found that torpid *M. ricketti* had lower levels of B6 and B12 (vitamin B6: 4.09 ± 0.08 ng/g; vitamin B12: 15.03 ± 1.11 ng/g) compared to non-torpid *M. ricketti* (vitamin B6: *P* < 0.001; vitamin B12: *P* < 0.001) (Table 1). There was no difference in the folate concentration in both groups of *M. ricketti* (torpid: 194.95 ± 3.75 ng/g; active: 191.46 ± 8.17 ng/g) (Table 1). 

**Table 1:** Concentrations of vitamin B6, vitamin B12 and folate in the brain.

	**Concentration (ng/g)^c^**		
**Animal group^a,^**^b^**	**Vitamin B6**	**Vitamin B12**	**Folate**
***M. ricketti* (AF)**	4.80 ± 0.09	17.49 ± 0.38	194.95 ± 3.75
***M. ricketti* (H-NF)**	4.09 ± 0.08**^d^**	15.03 ± 1.11**^d^**	191.46 ± 8.17
***R. leschenaultii* (AF)**	5.60 ± 0.16	21.77 ± 0.75	224.34 ± 27.45
***R. norvegicus* (AF)**	4.80 ± 0.36	17.46 ± 0.30	197.48 ± 10.23

^a^ Three (n = 3) animals in each group were analysed.

^b^ AF and H-NF represent bats treated with food in the active state and non-food in the hibernation state, respectively.

^c^ Concentrations are mean ± SD.

^d^ Statistical significance (*P* < 0.001) was found between AF and H-NF groups of *M. ricketti*.

**Figure 2:**
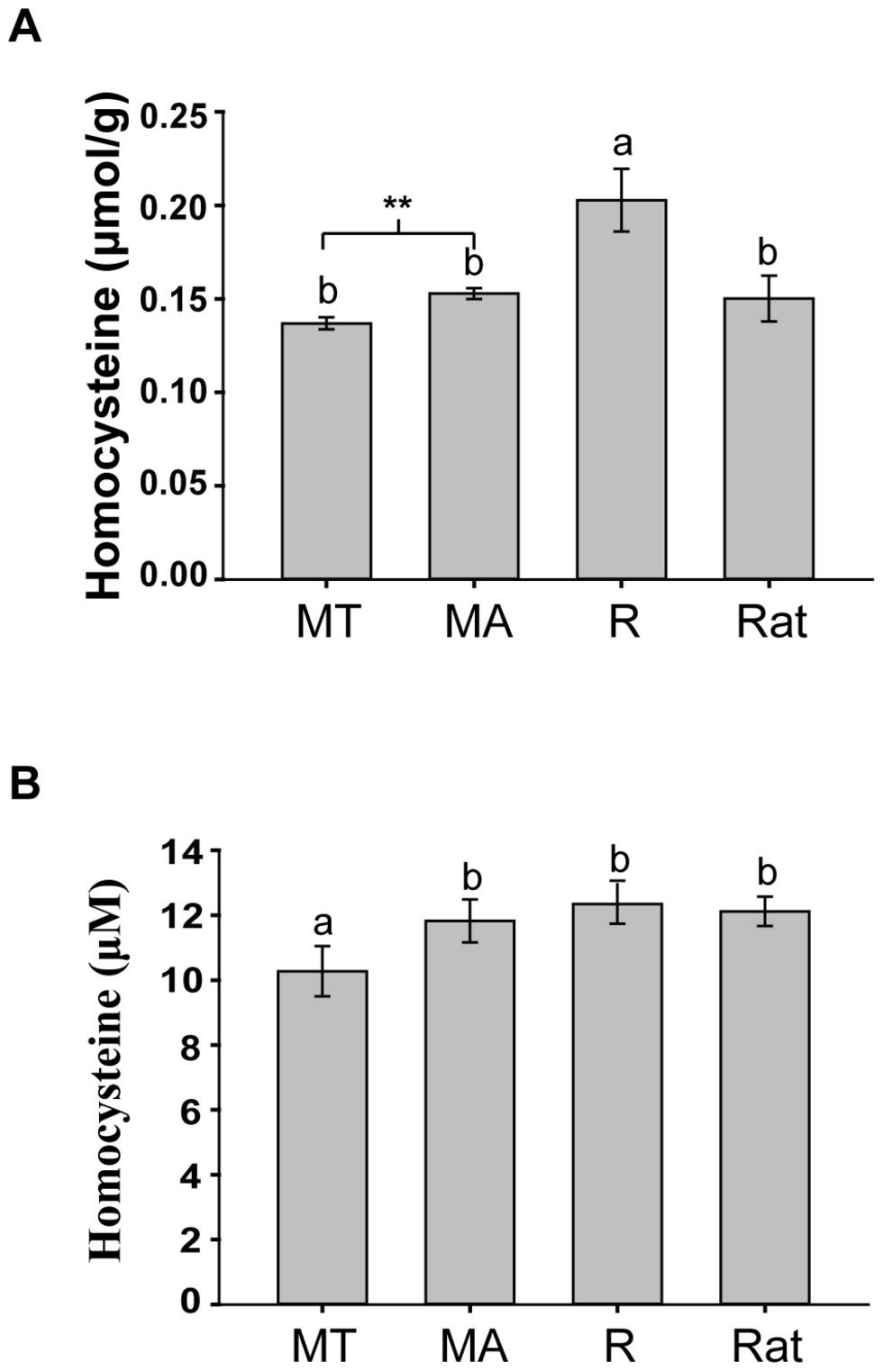
Expression levels of homocysteine in brain and plasma. Homocysteine expression levels were determined in the brains (A) and plasma (B) of torpid *M. ricketti* (MT), active *M. ricketti* (MA), *R. leschenaultii* (R) and rats. Data are expressed as mean ± SD from three separate experiments with n=3 per group. The letters (a,b) between groups represent statistical differences *P* < 0.001. ** denotes statistical significance (*P* < 0.001) between torpid and active *M. ricketti*.

Brain homocysteine levels in *M. ricketti* during torpor were approximately 10% lower than active *M. ricketti* (0.137 ± 0.003 vs. 0.153 ± 0.003 μmol/g, *P* < 0.001) (Figure 2A). Plasma homocysteine was also decreased in torpid *M. ricketti* compared to active *M. ricketti* (10.277 ± 0.774 vs. 11.826 ± 0.662μmol/L, *P* < 0.001) (Figure 2B). No difference was found between active *M. ricketti* and rats (0.150 ± 0.013 μmol/g, 12.122 ± 0.453 μmol/L) in brain tissue and blood. Homocysteine levels in *R. leschenaultia* (0.203 ± 0.017 μmol/g) were approximately 33–48 % higher than torpid and active *M. ricketti* and rats in the brain (*P* < 0.001), but there was no significant difference in plasma homocysteine among *R. leschenaultia* (12.345 ± 0.604 μmol/L), active *M. ricketti* and rats.

**Figure 3:**
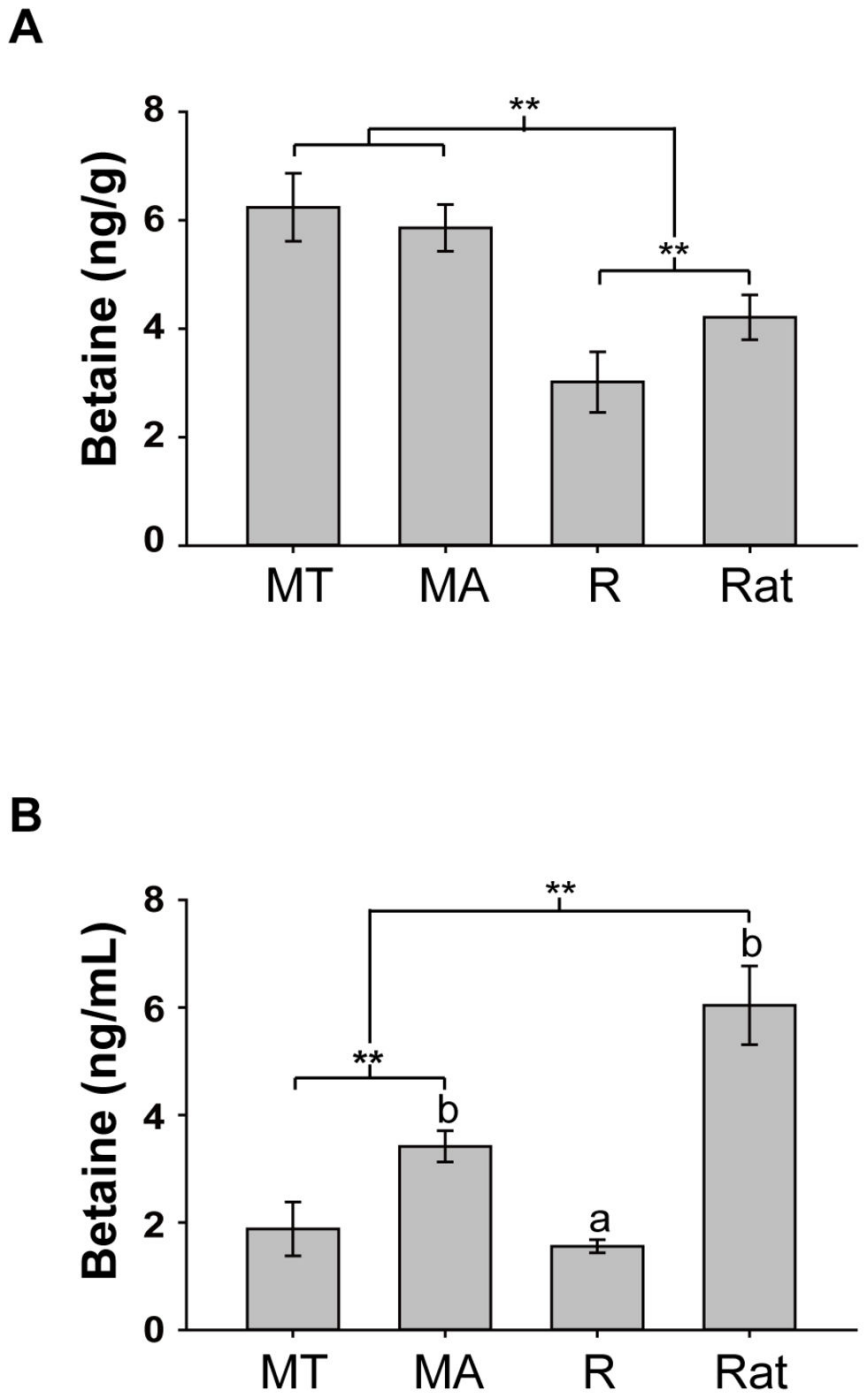
Betaine levels in brain and plasma. Brain betaine (A) and plasma betaine (B) were tested in torpid *M. ricketti* (MT), active *M. ricketti* (MA), *R. leschenaultii* (R) and rats. Three (n = 3) animals in each group were analysed. Results are expressed as mean ± SD of three repeats. The letters (a,b) between groups represent statistical differences *P* < 0.001. ** denotes statistical significance (*P* < 0.001) between groups.

The expression pattern of betaine was different between brain tissue and plasma (Figure 3A, B). Brain betaine levels in torpid *M. ricketti* (6.239 ± 0.626 ng/g) were similar to content in active animals (5.859 ± 0.430 ng/g), but torpid *M. ricketti* had lower levels of plasma betaine than active *M. ricketti* (1.879 ± 0.501 vs. 3.415 ± 0.292 ng/mL, *P* < 0.001). Betaine levels in the brains of active *M. ricketti* were higher compared to the rat brain (4.212 ± 0.413 ng/g, *P* < 0.001), but betaine levels in plasma of *M. ricketti* were lower than rats (6.042 ± 0.731 ng/mL, *P* < 0.001). Betaine levels of *R. leschenaultia* in the brain (3.015 ± 0.560 ng/g) and plasma (1.556 ± 0.122 ng/mL) were lower than active *M. ricketti* and rats (*P* < 0.001). 

### Western blot validation of enzymes involved in homocysteine metabolism

**Figure 4:**
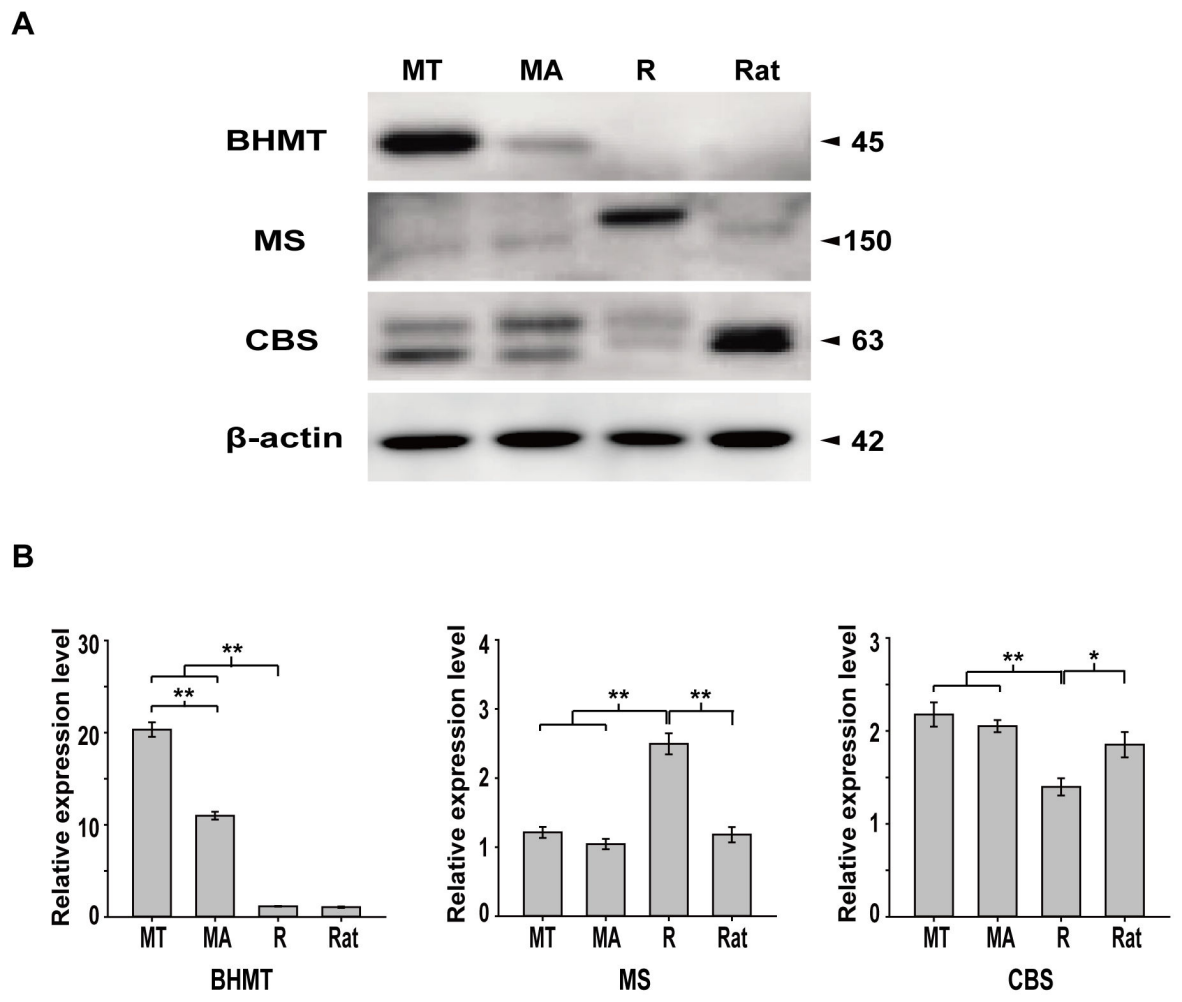
Western blot analysis. Expression levels of BHMT, MS and CBS were determined by Western blot (A). MT, torpid *M. ricketti*; MA, active *M. ricketti*; R, *R. leschenaultii*. All samples are from brain tissue. Arrows indicate predicated molecular weight (kDa) of proteins. (B) Relative expression levels of the proteins are represented as mean ± SD. Statistical significance by two tailed Student’s t-tests: *P<0.05, **P<0.001.

The expression levels of BHMT, MS and CBS were investigated in the brain of hibernating bats (Figure 4A). BHMT levels were 1.85-fold higher in torpid *M. ricketti* compared to active *M. ricketti* (Figure 4B); BHMT was not expressed in non-hibernating *R. leschenaultia* and rats (Figure 4A). There was no difference in total expression level of MS and CBS of torpid and active *M. ricketti*, and levels were similar to those in rats. Two clear bands of CBS near 63 kDa were detected and the amount of the bands showed reciprocal expression patterns between torpid and active states of *M. ricketti* bats. In *R. leschenaultia*, MS expression levels were higher and CBS expression levels lower compared to *M. ricketti* and rats (Figure 4B).

### BHMT activity assay

**Figure 5:**
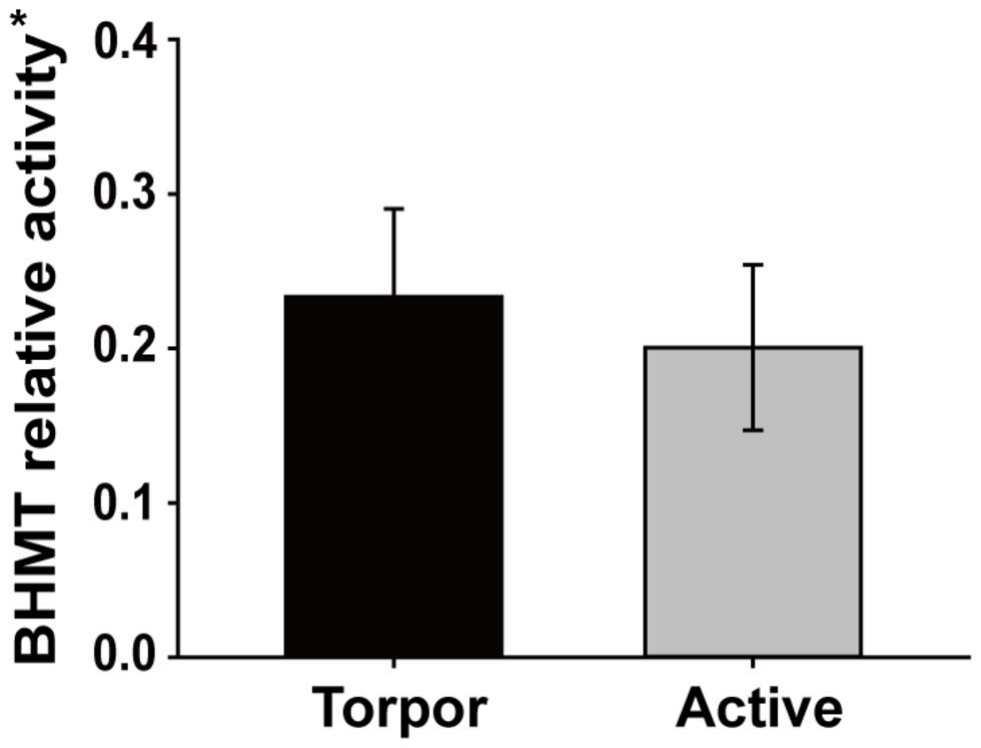
BHMT activity assay. The production amount of methionine represents BHMT relative activity. All results (mean ± SD) are from three separate experiments with n = 3 per group. Torpid and active states of *M. ricketti* are tested. Statistical significance is determined by Student’s t-tests (two tailed). * denotes nmol/h/mg protein.

More methionine was produced in torpid *M. ricketti* compared to active *M. ricketti* (0.432 ± 0.105 vs. 0.201 ± 0.054 nmol/h/mg protein) in brain tissue. Relative activity of BHMT was calculated by dividing the production amount of methionine by the relative expression level of BHMT, and revealed that there was no significant difference in BHMT relative activity between torpid and active *M. ricketti* (0.233 ± 0.057 vs. 0.201 ± 0.054 nmol/h/mg protein) (Figure 5). In addition, we noticed that the product level was lower in brain tissue compared to liver tissue [36], which may be due to BHMT expression levels in the brain being at least 100-fold lower than in the liver (unpublished data). 

### BHMT immunohistochemistry

**Figure 6:**
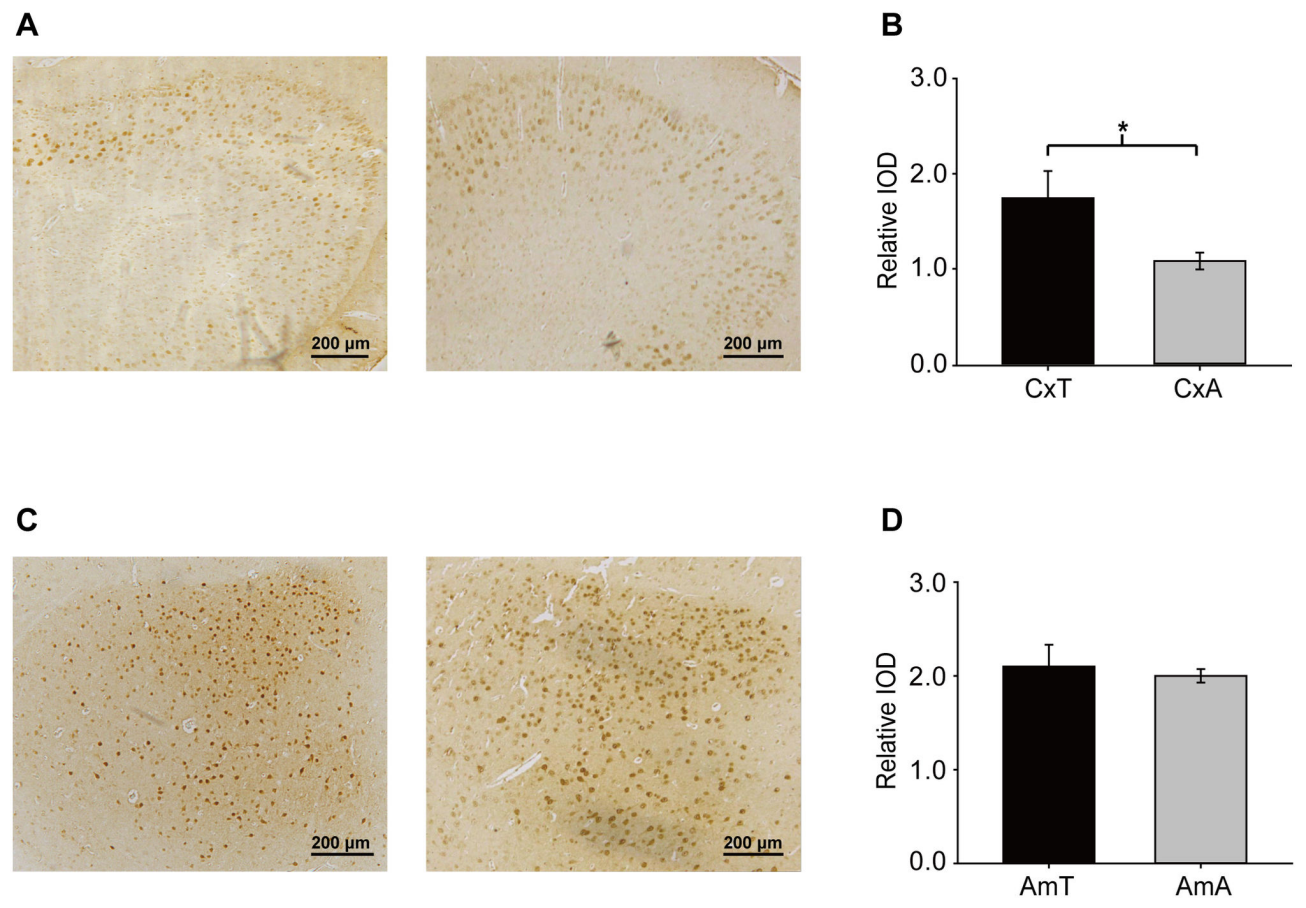
Immunohistochemical detection of BHMT in the brains of *M. ricketti*. After series of coronal sections, BHMT expression was identified in the cerebral cortex (Cx) and the amygdala (Am) of basal ganglia between torpid and active states in *M. ricketti*. Immunopositivity was dark-brown in the cytoplasm of cells. The four photomicrographs indicate encephalic regions and states: cerebral cortex, torpid state (A, left lane); cerebral cortex, active state (A, right lane); amygdala, torpid state (C, left lane); amygdala, active state (C, right lane). Quantitative analysis of BHMT immunostaining in cerebral cortex (B) and amygdala (D). Three (n = 3) animals in each group were analysed. Relative integrated optical densities are shown as mean ± SD. *P < 0.05 (two tailed Student’s t-tests). CxT, the cerebral cortex of torpid *M. ricketti*; CxA, the cerebral cortex of active *M. ricketti*; AmT, the amygdala of torpid *M. ricketti*; AmA, the amygdala of active *M. ricketti*.

MS and CBS is usually expressed in mammalian brains [3,43], and the expression levels in the brain tissues of *M. ricketti* were found to be similar to that in rats. However, BHMT is only expressed in *M. ricketti* brains, and previous experiments showed that BHMT is mainly detected in the liver and kidney [19]. To identify the distribution of BHMT in neuroanatomical locations that may be involved in the hibernation process, brain tissues from torpid and active *M. ricketti* were stained for BHMT expression. We found that BHMT was abundantly expressed in the telencephalon, including some regions of the cerebral cortex (such as the retrosplenial granular cortex, retrosplenial agranular cortex, primary motor cortex, secondary motor cortex, medial parietal association cortex, lateral parietal association cortex and primary somatosensory cortex, hindlimb region) (Figures 6A) and the amygdala of basal ganglia (Figures 6C). Integrated optical density of BHMT staining was increased in the cerebral cortex and no significant change was found in the amygdala of torpid *M. ricketti* (Figure 6B, D). 

### Selection pressure on *Bhmt* and structure analysis

**Figure 7:**
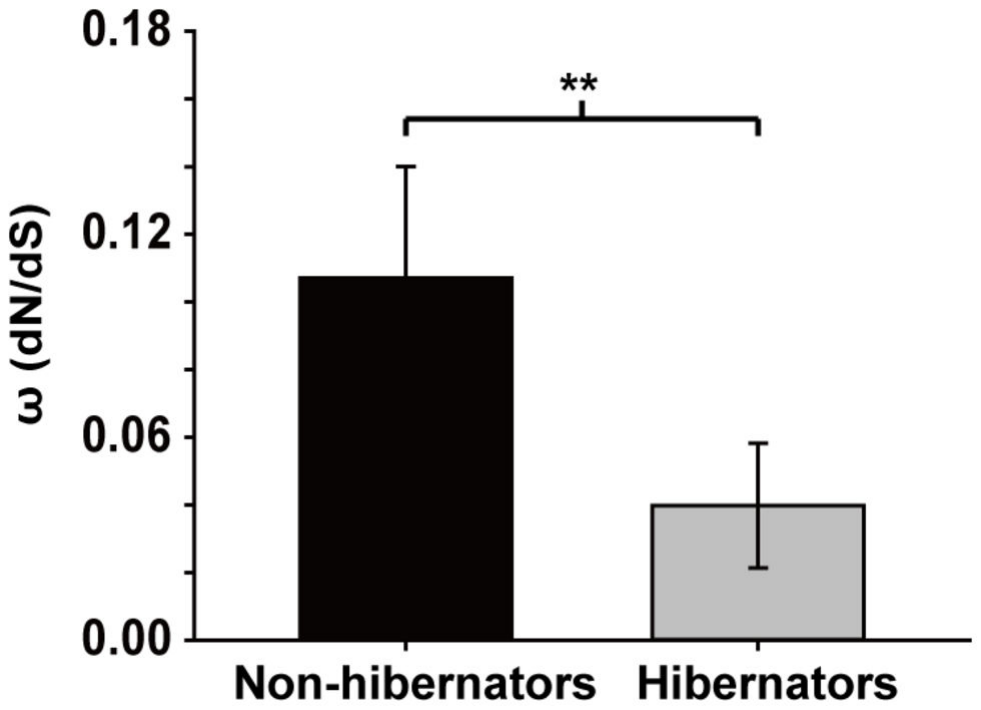
Selective pressure on *Bhmt*. Selective pressure on BHMT was determined by comparing the ω value (ω=dN/dS, dN: non-synonymous substitution rate, dS: synonymous substitution rate) for several species of non-hibernating (black bar) and hibernating (gray bar) bats. Data are presented as mean ± SD. Statistical significance was determined by Student’s t-tests (two tailed), **P < 0.001.

To investigate the evolution track of BHMT, the selection pressures on *Bhmt* of hibernating and non-hibernating bats was calculated. The encoding regions of *Bhmt* were cloned, sequenced and analysed from four non-hibernating and ten hibernating species of bat to determine *Bhmt* adaptation to hibernation (Table S1). To compare differences in selection pressure, we divided sequences into two groups (non-hibernation and hibernation) based on clear physiological characteristics (Figure S1 and Table S1). We found that BHMT is under strong purifying selection and the ω (dN/dS) value was lower than 0.18. A stronger selection constraint was found in hibernators than non-hibernators (Figure 7). 

**Figure 8:**
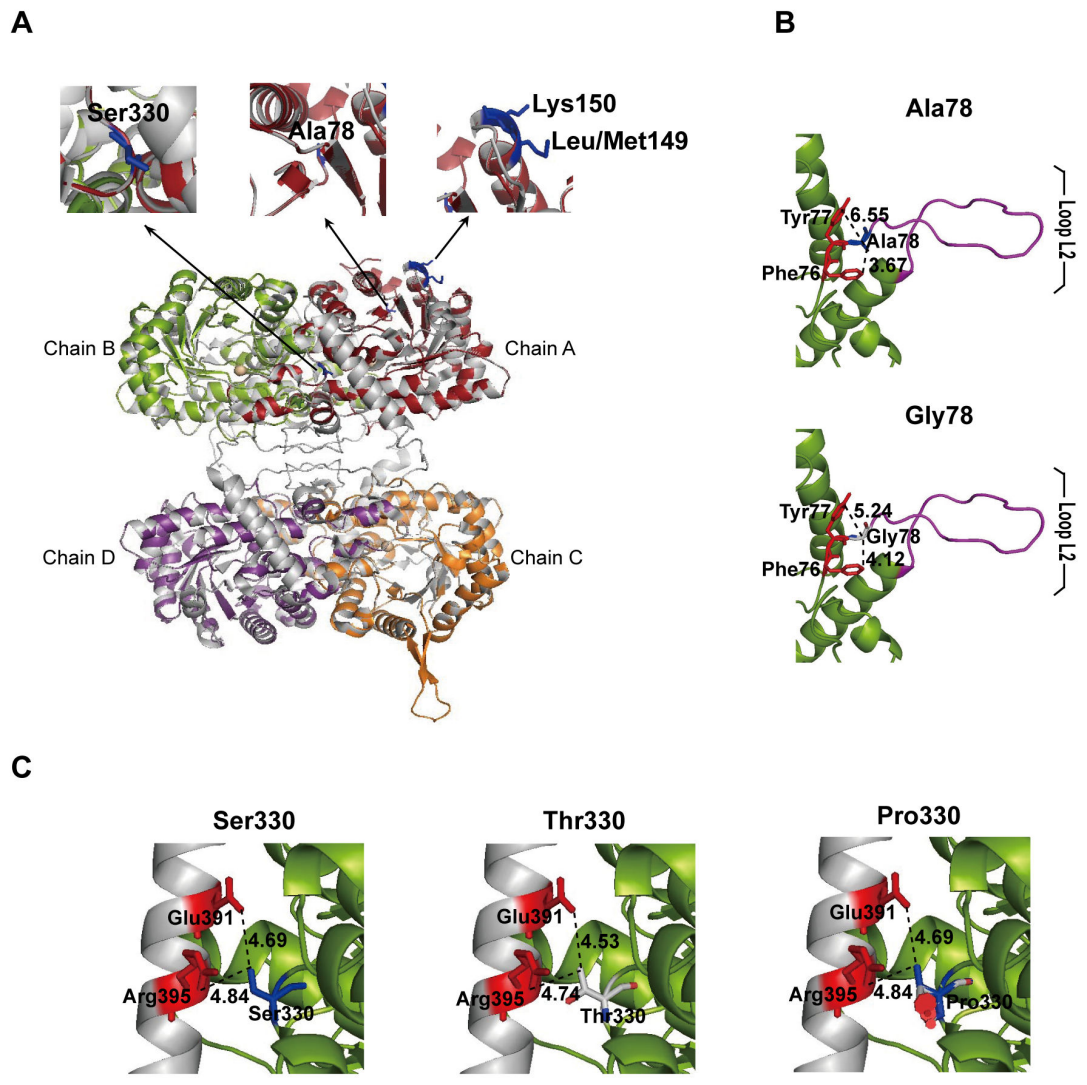
Homology modeling of bat BHMT. (A) BHMT tetramer of bat consists of chain A (red), B (green), C (orange) and D (purple). Bat BHMT was superposed to the 3D-structure of BHMT of rat (*Rattus norvegicus*, O09171) (gray). Four amino acid residues (Ala78, Leu/Met149, Lys150 and Ser330) that are conserved in all hibernators of bats but are varied among non-hibernators of bats are shown in blue. (B) Amino acid residue at position 78 linking the residues Phe76 and Tyr77 for substrate binding and loop L2 (residues 79–95) is Ala78 in hibernating bats (upper lane) but is Gly78 in non-hibernating bats (lower lane), in which the side chain distances between positions 78 and their adjacent residues Phe76 and Tyr77 are rearranged. (C) Ser330 in hibernating bats is different from Thr330 or Pro330 in non-hibernating bats, which may influence binding of the dimers AB to CD, especially the substitution of Pro330. Glu391 and Arg395 of chain D were stacked to the segment 316–349 of chain B, and dotted lines indicate the distance from Glu391/Arg395 to residues at position 330. All structures are represented by PyMol.

We aligned corresponding amino acid sequences of BHMT on the basis of required sequences (Figure S1). This showed high similarity between BHMT in the bat and rat whereby *M. ricketti* BHMT had a 96% similarity to rat BHMT (Figure 8A, Figure S1). Homology modeling revealed bat BHMT is composed of four identical subunits, which consisted mostly of a (α/β)_8_ barrel (Figure 8A) [44]. Most amino acids were conserved in bats, but four amino acid sites (at positions 78, 149, 150 and 330) were different between non-hibernating and hibernating bats (Figure 8). Position 78 was changed from Ala in hibernating bats to Gly in non-hibernating bats (Figure 8B). Position 149 and 150 locating in α3 helix were variable from Leu/Met and Lys to Ile and Arg, respectively. Ser330 was situated in segment (residues 316-349) in hibernating bats, and was substituted by Thr330 or Pro330 in non-hibernating bats (Figure 8C). 

## Discussion

This study confirms that homocysteine does not accumulate in brain tissue of torpid *M. ricketti*, despite declining B vitamin levels from fasting. One reason for this pattern could be that the key catalyzing homocysteine enzymes BHMT, MS and CBS do not decline during hibernation. Another explanation is the role of other molecules in the control of hibernation. For example, one of the products of homocysteine metabolism, methionine, is an essential amino acid in mammals because it is not synthesized *de novo* [3]. Methionine and its metabolic products are involved in multiple fundamental biological processes (Figure 1) [43] and methionine is catalized to become S-adenosylmethionine (SAM, an important methyl donor) by methionine adenosyltransferase (MAT). Several studies have demonstrated that the transcription and translation of many genes are regulated by methylation during mammalian hibernation. Methylated DNA has a strong impact on gene expression [24]. Hibernation-nonresponsive genes show enhanced methylation of their promoter regions to switch off transcription [24]. In addition, some enzymes, such as protein phosphatase 2A (PP2A) involved in tau phosphorylation in hibernation, can be activated by methylation of its catalytic subunit [45]. Therefore, methylation may require more SAM, which promotes homocysteine methylation and results in a decrease in homocysteine in brain tissue. 

The amount of homocysteine in the brain of non-hibernating *R. leschenaultia* is higher than for *M. ricketti* and rats. It is possible that the normal range of homocysteine is wider in *R. leschenaultia* or there exists other mechanisms to prevent homocysteine injury. BHMT was not detected in the brains of *R. leschenaultia* and rats, but MS is higher in *R. leschenaultia* brain tissue than *M. ricketti*, suggesting that MS is crucial to the homocysteine methylation pathway when BHMT is absent. In addition, we found that brain homocysteine, MS and CBS levels in torpid *M. ricketti* were similar to rats (Figures 2, 4) except for BHMT expression. Whether the normal range of homocysteine homeostasis in hibernating bats can be judged using parameters from rats needs to be verified.

Hyperhomocysteinemia has been diagnosed based on the plasma homocysteine concentration in clinical medicine [9]. In this study, we found that plasma homocysteine is not increased in *M. ricketti* during hibernation and indicate that homocysteine concentration is maintained at a normal level in blood and brain tissues regardless of whether *M. ricketti* hibernates, which is beneficial to protect hibernators from atherothrombosis and thrombosis. The amount of plasma homocysteine in non-hibernating *R. leschenaultia* is close to active *M. ricketti* and rats, which is different from the condition in the brain between these species. The data reported here suggest that homocysteine levels are not consistent between blood and local tissue, and it is necessary to measure homocysteine in both.

Moreover, BHMT expression is elevated in the brains of torpid *M. ricketti*, compared to active animals, and its relative activity does not differ across the two states. The BHMT/betaine system exhibits a protective effect from homocysteine-induced injury [46] and BHMT deficiency leads to the elevation of total homocysteine concentration in *Bhmt* ^*-/-*^ mice [47]. BHMT is a key modulator of homocysteine when expression or activity of other enzymes is abnormal [48,49]. The inherent kinetic properties of the enzyme are likely important [43] and compared with MS and CBS, BHMT has a lower K_m_ for sulfur-containing substrates [43]. BHMT can utilize homocysteine at relatively low concentrations and has a high affinity for homocysteine. Elevated BHMT in the brain during hibernation strongly suggests that BHMT play a role in the regulation of homocyteine during this state.

Hibernation inhibits betaine sourced from food and overall reduced metabolism results in a decrease in plasma betaine during hibernation (Figure 3B). However, brain betaine levels were not different between torpid and active *M. ricketti* (Figure 3A). The physiologic function of betaine is either as the methyl donor involved in BHMT-catalyzed homocysteine metabolism or as an osmolyte protecting cells, proteins and enzymes from environmental stress (such as extreme temperature in hibernation) [50]. The difference in betaine levels in the brain and plasma suggests that hibernators adjust the betaine distribution for homocysteine metabolism and brain protection. Furthermore, several studies have reported that the synthesis and reserves of acetylcholine are lower during hibernation than when active [51]. Acetylcholine (as a neurotransmitter) may be broken down to choline and oxidized to betaine when nerve conduction is reduced during hibernation [19,22]. Therefore, BHMT-catalyzed reactions play an important role in betaine homeostasis in addition to homocysteine levels. 

The total amount of MS and CBS did not vary between torpid and active *M. ricketti*, but their function could be influenced during hibernation. B vitamin supplementation (vitamin B6, vitamin B12 and folate) and strengthening of the transulfuration and transmethylation pathways could lower homocysteine levels and slow the accelerated rate of neurological disease [12,52,53]. However, B vitamins are not synthesized by hibernators themselves, and fasting during hibernation leads to reduced intake of vitamin B6 and vitamin B12 in torpid bats. The brain is not the main storage organ of B vitamins in bats [54], and they are not quickly transported to the brain because of the lower flow velocity of blood [32,55]. These factors may inhibit the involvement of MS and CBS in transmethylation and transsulfuration. Meanwhile, the transsulfuration pathway is inactivated when methyl transfer (such as the process involving SAM) requirements are high [56], consistent with the idea that homocysteine levels are usually determined by remethylation rather than transsulfuration during fasting [57,58]. Interestingly, following declining vitamin B6 and vitamin B12, the level of folate did not vary between torpid and active bats (Table 1). This observation could be explained by the “methyl trap” considering that vitamin B12 deficiency reduces the activity of MS. It leads to the accumulation at other folate forms [59,60] and could inhibit folate converting to tetrahydrofolate (THF). These processes may discount the roles of MS and CBS during hibernation and make the role of BHMT more important in homocysteine homeostasis. However, two bands of CBS detected in *M. ricketti* bats exhibit a reciprocal expression pattern between torpid and active states (Figure 4A), which suggests the occurrence of post-translational modifications (PTMs) on CBS. PTMs (e.g., sumoylation and methylation) are known to be intensively involved in regulation of CBS activities [61–64]. Whether catabolism of homocysteine by transsulfuration pathway is reduced in torpid bats due to decreased B vitamins and PTMs of CBS remains to be investigated. Thus, the increased level of BHMT, reduced activity of transsulfuration pathway, and lower production of homocysteine could together maintain the homocysteine level during bat torpor. 

Immunocytochemistry revealed that the cerebral cortex and the amygdala of basal ganglia were the main encephalic regions of BHMT distribution. Elevated homocysteine levels may result in the formation of neurofibrillary tangles (NFTs), which are severe in the cerebral cortex and amygdala in AD patients [65]. BHMT expression in these two regions may modulate homocyteine levels and decrease production of homocystine-induced NFTs in order to prevent relative neurological damage during hibernation.

Furthermore, *Bhmt* is found under strong purifying selection (ω < 0.18) and there exists a stronger selective constraint in hibernating bats (Figure 7), indicating that this gene is very conservative and reflects a higher functional constraint on BHMT protein [33]. The significant difference in selective pressure between hibernators and non-hibernators may be the result of environmental stressors, such as low temperature and food shortage [33]. This analysis supported the functional importance of BHMT during hibernation. 

 The functional importance of BHMT in control of homocysteine levels in the brain of torpid bats is further supported by the observation that the amino acid residues of BHMT is more conserved in hibernating bats than that in non-hibernating bats (Figure 8). Three (positions 78, 150 and 330) of four different sites (positions 78, 149, 150 and 330) were 100% conserved in all ten hibernating bats but were diverged in four non-hibernating bats compared to other mammal species (Figure S1). Amino acid Ala78 links loop L2 (residues 79–95) and residues Phe76 and Tyr77 that are critical for substrate binding (Figure 8B) [44,66]. Phe76 has been included in the betaine-binding site and involved in properly orienting the Hcy, and Tyr77 is contributing to the hydrophobic core for betaine and provides a hydrogen bond for betaine [44]. On the other hand, loop L2 presents mobility by playing an essential role in Hcy binding [44]. Therefore, Ala78 as a linker between the two regions may play a role to influence substrate binding. Amino acid site 330 located in segment 316–349 (chain B) is stacked to a helix (residues 381–407) of the interacting subunit (chain D), which contributes to the tetramerization of AB and CD [44]. The amino acid change at position 330 may influence BHMT structural stability, especially when Ser330 was substituted by Pro330 (Figure 8C). The substitutions at positions 78 and 330 change the side chain distance between these residues and their adjacent amino acids, whether the alteration of van der Waals’ forces among the residues affects BHMT function remains to be investigated (Figure 8B, C). 

We found for the first time that BHMT is expressed in the brains of mammals. Expression in the cerebral cortex and amygdala, and strong purifying selection in BHMT, suggest that hibernators regulate BHMT expression to decrease homocysteine-induced brain damage. This research builds on our understanding of homocysteine homeostasis and this is essential to develop treatments for human cerebrovascular and neurodegenerative diseases. It is clear that BHMT is important to homocysteine homeostasis in brain tissue and the distribution and function of BHMT in other tissues and species is an exciting area of further research.

## Supporting Information

Figure S1 **Alignment of amino acid sequences of BHMT.** Amino acid sequences ranging from 22 to 354 were aligned from bats and human. Amino acid site numbers are referenced to mature human BHMT. The amino acid sites indicated by asterisk (*) mean that the corresponding amino acids are different between hibernating (H) bat group and non-hibernating (N) bat group.
(TIF)

Table S1 **The accession numbers and heterothermy condition in bat species.**
(DOCX)

Table S2 **Primers used for amplification of *Bhmt* gene from 12 bat species.**
(DOCX)
