# Supplementary figures and images for: Homocysteine Homeostasis and Betaine-Homocysteine S-Methyltransferase Expression in the Brain of Hibernating Bats
Source: PLoS One. 2013 Dec 23;8(12):e85632. doi: 10.1371/journal.pone.0085632 (PMC3871600; doi:10.1371/journal.pone.0085632)

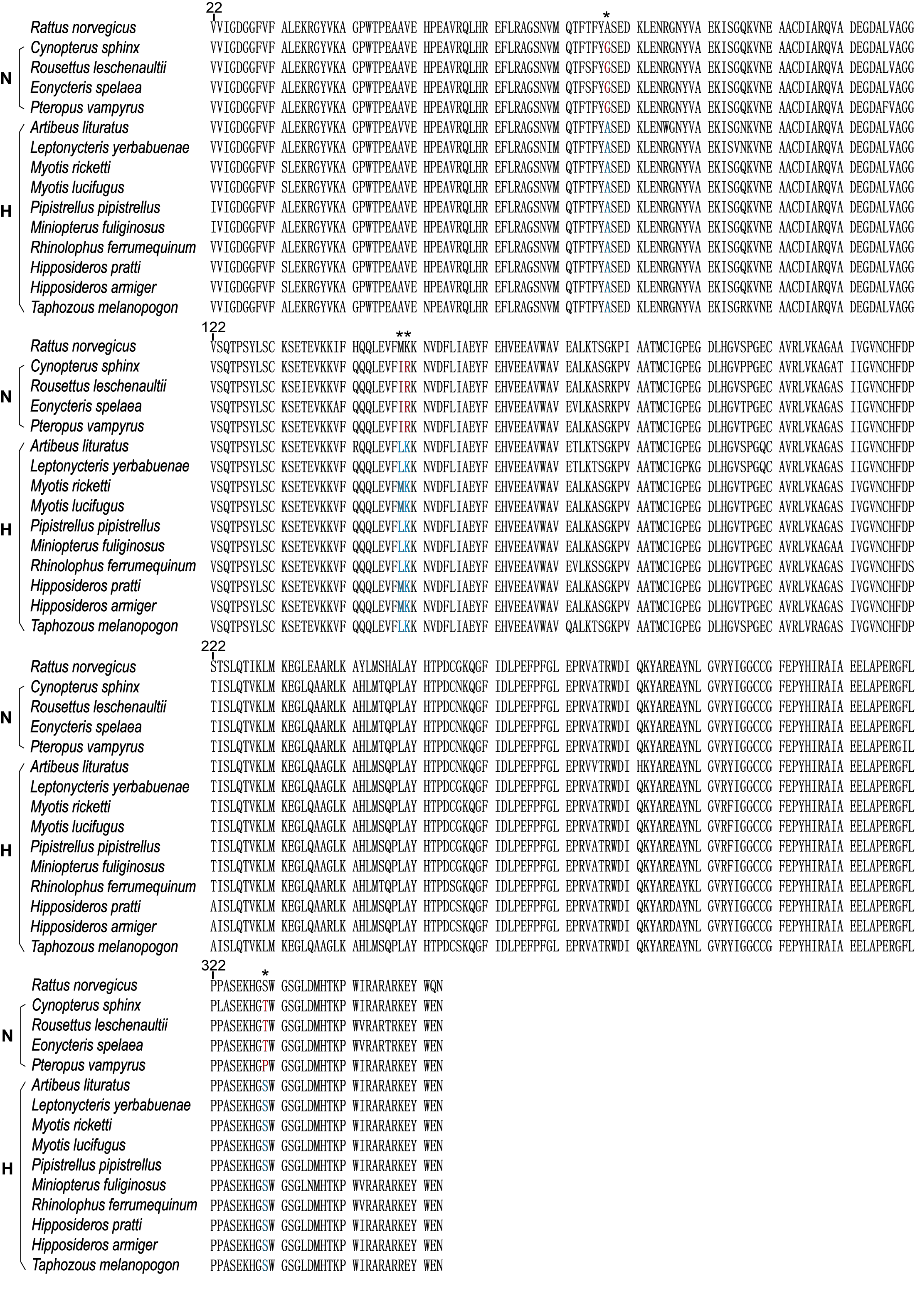

Supplement: Figure S1 — Alignment of amino acid sequences of BHMT. Amino acid sequences ranging from 22 to 354 were aligned from bats and human. Amino acid site numbers are referenced to mature human BHMT. The amino acid sites indicated by asterisk (*) mean that the corresponding amino acids are different between hibernating (H) bat group and non-hibernating (N) bat group. (TIF) [file pone.0085632.s001.tif]
