# Supplementary material for: Homocysteine Homeostasis and Betaine-Homocysteine S-Methyltransferase Expression in the Brain of Hibernating Bats
Source: PLoS One. 2013 Dec 23;8(12):e85632. doi: 10.1371/journal.pone.0085632 (PMC3871600; doi:10.1371/journal.pone.0085632)
Supplement: Table S1 — The accession numbers and heterothermy condition in bat species. (DOCX) [file pone.0085632.s002.docx]

**Table S1. The accession numbers and heterothermy condition in bat species.**

| **Species** | **Accession Numbers** | **Heterothermy condition** |
| --- | --- | --- |
| **Family Pteropodidae** |  |  |
| *Eonycteris spelaea* | KF051787 | N |
| *Rousettus leschenaultii* | KF051795 | N |
| *Cynopterus sphinx* | KF051786 | N |
| *Pteropus vampyrus* | ENSPVAG00000000624 | N |
| **Family Emballonuridae** |  |  |
| *Taphozous melanopogon* | KF051785 | H |
| **Family Miniopteridae** |  |  |
| *Miniopterus fuliginosus* | KF051791 | H |
| **Family Vespertilionidae** |  |  |
| *Pipistrellus pipistrellus* | KF051793 | H |
| *Myotis ricketti* | KF051792 | H |
| *Myotis lucifugus* | ENSMLUG00000009164 | H |
| **Family** [**Phyllostomidae**](http://animaldiversity.ummz.umich.edu/site/accounts/classification/Phyllostomidae.html) |  |  |
| *Artibeus lituratus* | KF051784 | H ? |
| *Leptonycteris yerbabuenae* | KF051790 | H ? |
| **Family Hipposideridae** |  |  |
| *Hipposideros armiger* | KF051788 | H |
| *Hipposideros pratti* | KF051789 | H |
| **Family Rhinolophidae** |  |  |
| *Rhinolophus ferrumequinum* | KF051794 | H |

H: Hibernating bat.

N: Non-hibernating bat.

?: It is likely torpor, but no references.
