# Supplementary material for: Homocysteine Homeostasis and Betaine-Homocysteine S-Methyltransferase Expression in the Brain of Hibernating Bats
Source: PLoS One. 2013 Dec 23;8(12):e85632. doi: 10.1371/journal.pone.0085632 (PMC3871600; doi:10.1371/journal.pone.0085632)
Supplement: Table S2 — Primers used for amplification of Bhmt gene from 12 bat species. (DOCX) [file pone.0085632.s003.docx]

**Table S2.** **Primers used for amplification of *Bhmt* gene from 12 bat species.**

| **Primer sequence** | | **Tm (^o^C)** | **Species** | **Product length (bp)** |
| --- | --- | --- | --- | --- |
| F1  R1 | 5'-GCCAAGAAGGGCATCCTAGAACG-3'  5'-AGATTCTCCCAGTATTCCTTCCTG-3' | 53°C | *Rousettus leschenaultia*  *Cynopterus sphinx* | 1040 |
|  |  |  | *Taphozous melanopogon* |  |
|  |  |  | *Miniopterus fuliginosus* |  |
|  |  |  | *Pipistrellus pipistrellus* |  |
| F1 | 5'-GCCAAGAAGGGCATCCTAGAACG-3'  5'-CTTTGAGATTGAAGGGTTGTATGG-3' | 53°C | *Myotis ricketti*  *Hipposideros armiger*  *Hipposideros pratti*  *Rhinolophus ferrumequinum*  *Eonycteris spelaea*  *Artibeus lituratus*  *Leptonycteris yerbabuenae* | 1083 |
| R2 |  |  |  |  |
